# Pregnant adolescents’ lived experiences and coping strategies in peri-urban district in Southern Ghana

**DOI:** 10.1186/s12889-022-13318-2

**Published:** 2022-05-05

**Authors:** Agnes M. Kotoh, Bernice Sena Amekudzie, Kwabena Opoku-Mensah, Elizabeth Aku Baku, Franklin N. Glozah

**Affiliations:** 1 School of Public Health, College of Health Sciences, University of Ghana, P.O. Box LG 13, Legon, Accra, Ghana; 2 School of Nursing and Midwifery, University of Health and Allied Sciences, PMB 31, Ho, Volta Region Ghana

**Keywords:** Adolescent pregnancy, Sexual abuse, Stigma, Coping strategies, Perversion of justice, Ghana

## Abstract

**Background:**

Adolescence, a transition period from childhood to adulthood forms the foundation of health in later life. The adolescence period which should have been characterised by good health is often marred with life-threatening and irreparable consequences of public health concern. Teen pregnancy is problematic because it could jeopardise adolescents’ safe transition to adulthood which does not only affect adolescents, but also their families, babies and society. There is ample evidence about the determinants and effects of teen pregnancy, but it is fragmented and incomplete, especially in Sub-Sahara Africa. This study presents pregnant adolescents’ voices to explain significant gaps in understanding their lived experiences and coping strategies.

**Methods:**

This narrative inquiry, involved in-depth interviews with 16 pregnant adolescents, who were recruited from a peri-urban district in Southern Ghana using purposive and snowball techniques in health facilities and communities respectively. The audio recorded interviews were transcribed verbatim and analysed manually using content analysis.

**Results:**

Many pregnant adolescents are silent victims of a hash socio-economic environment, in which they experience significant financial deprivation, parental neglect and sexual abuse. Also, negative experiences of some adolescent girls such as scolding, flogging by parents, stigmatisation and rejection by peers and neighbors result in grieve, stress and contemplation of abortion and or suicide. However, adolescents did not consider abortion as the best option with regard to their pregnancy. Rather, family members provided adolescents with critical support as they devise strategies such as avoiding people, depending on God and praying to cope with their pregnancy.

**Conclusion:**

Adolescent pregnancy occurred through consensual sex, transactional sex and sexual abuse. While parents provide support, pregnant adolescents self-isolate, depend on God and pray to cope with pregnancy and drop out of school. We recommend that the Ministries of Education and Health, and law enforcement agencies should engage community leaders and members, religious groups, non-governmental organisations and other key stakeholders to develop interventions aimed at supporting girls to complete at least Senior High School. While doing this, it is also important to provide support to victims of sexual abuse and punish perpetrators accordingly.

## Background

Adolescence, a transition period from childhood to adulthood forms the foundation of health in later life [1]. Unfortunately, the increasing adolescent population, the largest in history, which should have been characterised by good health is marred with sexual and reproductive health (SRH) challenges of public health concern especially in low- and middle-income countries (LMICs) [2–8]. The UNFPA reports 18.8% prevalence of adolescent pregnancy in Africa [9]. The 2014 Ghana Demographic Health Survey (GDHS) shows that 31.4% of adolescents had a child by age 19 [10]. Also, 14% of adolescents aged 15–19 years contribute to 30% of all deliveries in 2014 [4, 7].

Teenage pregnancy, mostly unplanned, is a social problem. It truncates adolescents’ childhood and jeopardizes’ their right to a safe transition into adulthood before they are developmentally, emotionally and socially ready [6, 11, 12]. The transition to motherhood needs physical, psychological, social and cognitive preparedness; but most teenagers take up the role of nurturing babies inadequately prepared [3, 4, 7, 11]. The teenager has to deal with the unexpected demands of being an adult, disapproval and disappointment shown by parents and relatives [6, 13, 14] and disruption of schooling, relationship problems with relatives, partners and peers [11, 15].

Studies in Sub-Saharan Africa and Southern Asia show that adolescent pregnancy is associated with unhealthy environment, low educational attainment and poverty [4, 16–18]; resulting in adverse health, economic and psychosocial outcomes with irreparable consequences of public health concern [5, 9–11, 19]. The adolescent, which may already be malnourished will have to share nutrients with the unborn child, often resulting in adverse outcomes [18]. In Ghana, maternal mortality rate is higher among 12–19 year olds (679 per 100,000 live births) compared to 380 and 359 per 100,000 live births among adults aged 20–24 and 25–29 years respectively [10].

There is ample evidence about the determinants and effects of teen pregnancy, but it is incomplete, especially in Sub-Sahara Africa. Several studies in sub–Saharan Africa and Southern Asia have shown that adolescent pregnancy is associated with unhealthy childhood environment, socio-economic conditions, low educational attainment, poverty, peer pressure and gender issues [4, 13, 16–18]. Also, pregnant adolescents’ experiences are multi-dimensional, ranging from physical, psycho-social and economic [10, 20]. In Ghana, an environment that is full of sexual taboos and abstinence-only sex education, together with limited negotiation skills shape the sexual decisions and behaviours of adolescent girls. Furthermore, limited knowledge of contraception, low self-efficacy in obtaining contraceptive methods such as condoms, and lack of skills to negotiate condom use are associated with adolescent pregnancy [3, 21]. These notwithstanding, there are gaps in the literature regarding how they got pregnant and the coping mechanisms they used.

Also, pregnant adolescents’ voices that should flame public debate, draw public health advocates’ and policy makers’ attention to the subject are almost absent. A study of this nature is relevant in Ghana in the light of public health concerns arising from the increasing rate of suicidal ideation and behaviour among adolescents. Using a narrative approach, this study explored how adolescents get pregnant, their lived experiences, and coping strategies to inform interventions for challenges of the environment in which girls grow and ultimately reduce, if not eliminate adolescent pregnancy in Ghana and other LMICs.

## Methods

### Design

A narrative inquiry research design was used and it involved conducting in-depth interviews (IDIs) with pregnant adolescents who are resident in the Keta Municipal Area (KMA). This made it possible for the adolescents to give an account of their most profound experiences, stories, and narratives.

The KMA is located in the Volta Region along the eastern part of the Volta estuary. It is about 160 km from Accra the capital city of Ghana. Healthcare services are provided mainly by government, supported by Christian Health Association of Ghana (CHAG). There are 28 health facilities within the municipality. These comprise of two hospitals (one public and one CHAG), 13 health centres, four Community-based Health Planning and Services (CHPS) zones, five maternity homes and four private clinics.

### Participants

Sixteen pregnant adolescents were recruited using purposive and snowball sampling techniques. Purposive sampling was used to recruit six out of the eight pregnant adolescents who had attended antenatal care (ANC) clinic at the KMA Hospital within the month preceding the study. A research team member who conducted the interviews took their contacts from the ANC register and traced them to their communities with the help of community health nurses. They were informed about the study and recruited after consenting to participate in the study. The recruitment process took two weeks. At the end of each interview, participants were asked about other pregnant adolescents. Potential participants were contacted and traced to their residents. Those who met the eligibility criteria (i.e., not more than 19 years and resident in the district) were recruited.

### Data collection

Face-to-face in-depth interviews (IDIs) were conducted with pregnant adolescents using a pretested guide developed based on literature. Data collection which lasted two months was done by a research team member with Master of Public Health degree and experience in conducting in-depth interviews. The questions include information on their demographic profile, how they got pregnant, challenges they encountered and how they are coping with their situation. While fourteen interviews were conducted in Ewe (the local language) and two in English language. The interviews were conducted in conducive places, mainly at community centres and other places in the community of adolescents’ chioce.

A non-judgemental environment and dialogical approach during discussions encouraged participants to honestly tell their stories; describing how they got pregnant, their experiences and coping strategies. Drawing on the principles of saturation [22], data collection stopped at the 16^th^ interview when no new ideas or perspectives were emerging. The interviews were audio recorded, with each interview lasting for about 40 min.

### Data analysis

The data was analysed by the research team. The recorded discussions were transcribed verbatim and analysed manually using inductive content analysis approach. This involves reading transcripts thoroughly several times and condensing the raw textual data into a summary format [23]. Both similar and different views on the subject were grouped into themes and sub-themes and clear links established between findings and research objectives. This aided the comparison of various issues mentioned. Also, participants’ statements were used to support the themes generated and illuminate their perspectives.

### Trustworthiness

Trustworthiness is established when findings as much as possible reflect participants’ views [24]. Steps taken to ensure that the study is trustworthy include: building trust, notes written during the study were used to confirm participants’ responses, back translation method used to translate the interview guide and transcripts to ensure participants understand the questions as intended and their perspectives were not lost during the translation process. Also, the research team looked for verbatim quotes from participants’ narrations to support themes and subthemes generated and demonstrate issues paramount to them.

### Ethical issues

The Ghana Health Service (GHS) Ethical Review Committee gave ethical approval [GHS-ERC 023/06/19] for this study. All study procedures were performed in accordance with relevant ethical principles for medical research involving human subjects. Participating in the study was preceded by a written informed consent processes communicated to prospective participants. Participants gave consent after they were informed about the study’s aim (to explore how adolescents get pregnant, their lived experiences and coping strategies), and the freedom to decline the request to participate, refuse to answer any question as well as redraw from the study anytime they wish. Written informed consent was given by all participants 18-years and above and assent obtained from parents/guardians of those under 18 years for allowing their children to participate in the study and recording the interviews. Consent was sought before audio recording interviews. A counselor was engaged to attend to participants who might show austere emotional distress during or after the interviews. Interviews were conducted in places devoid of intruders, often community centres, school compound, churches and adolescents home depending on the preference of participants to ensure privacy. Anonymity and confidentiality were guaranteed by using pseudonyms during the interviews and presentation of the results.

## Results

### Socio-demographic characteristics of pregnant adolescents

The participants were aged between 12–19 years. Ten were in primary school. None were married and 14 Christians (Table 1).

**Table 1:** Socio-demographic characteristics of pregnant adolescents (*N* = 16)

Age	N	Who participants live with	N
12	1	Mother	6
13	1	Father	1
14	3	Grandmother	4
15	3	Aunt	1
16	2	Siblings	2
17	3	Partner	1
18	2	Partner’s mother	1
19	1		
**Educational level**		**Marital Status**	
None	1	Married	0
Primary	10	In relationship	7
Junior High School (JHS)	3	Not in relationship	9
Senior High School SHS)	2		
**Occupation**		**Religion**	
Students	14	Christianity	14
Apprentice	1	Traditionalists	1
Trading	1	None	1

Majority of pregnant adolescents’ fathers and mothers have no formal education. Three mothers and seven fathers attended primary school, two mothers and one father attended JHS. Only two fathers attained tertiary education. Also, majority of parents were self-employed. Only three fathers were formal sector employees (Table 2).

**Table 2:** Background characteristics of participants’ parents

Parents’ Background Characteristics	Mothers (*n* = 16)	Fathers (*n* = 14)
**Educational background**		
No formal education	9	7
Primary	5	2
JHS	2	1
SHS	-	2
Tertiary		2
**Occupation**		
Fisherman	-	7
Fishmonger	6	-
Trader	7	1
Farmer	1	2
Sailor	-	1
Policeman	-	1
Teacher	-	1
Unemployed	2	1

### Themes and sub-themes

Three main themes that emerged from the data are: how adolescents got pregnant, pregnant adolescents’ experiences and coping strategies (Table 3).

**Table 3:** Themes and Sub-themes

Themes	Sub-themes
**1. How adolescents got pregnant**	Consensual sex
	Sexual abuse
	Transactional sex
**2. Experiences of pregnant adolescents**	Psycho-social challenges
	Financial challenges
	Health challenges
	Stopped schooling
**3. Coping strategies**	Support from social network
	Avoidance of people
	Dependence on God and prayer

### How adolescents got pregnant

Of the 16 pregnant adolescents studied, six pregnancies occurred through consensual sex, five through abuse and five through transactional sex; with their ages ranging between 14–19, 12–16 and 14–17 years respectively.

#### Consensual sex

Participants who got pregnant through consensual sex gave the following narrations:

*He is my boyfriend. We slept together. I realised I was pregnant when I missed my menses for three months. I thought it was normal until my mum said I was pregnant. I denied but it was confirmed.* (14 years)

*My sister never allows me to go out before I met my boyfriend. He told my sister that he is dating me. We have been having sex. The pregnancy was unplanned.* (18 years)

#### Transactional sex

Participants who got pregnant through transactional sex, narrated how financial deprivation forced them to engage in sex for money and material things:

*I needed money to buy things for SHS. So, I started working in a shop. The owners’ son told me he could help me only if I had sex with him. I didn’t have any choice so I agreed. We started having sex and I became pregnant.* (17 years).

*My father left us. My mother was caring for us. Things were difficult, I couldn’t buy books and pay my fees. My dresses and shoes were worn out. But I loved going to school. So I started asking the boy for money. He gives me money and other things. One day he asked me to wash his things and we had sex. I agreed because I needed his help. It was my first time but I got pregnant.* (15 years).

#### Sexual abuse

Eleven of the 16 participants were sexually abused; usually by uncles, school mates and acquaintances. The three raped and eight defiled victims were aged 16–19 years and 12–15 years respectively. They could not report the incident immediately because of shyness and fear. Those who told their family members later were beaten and silenced or threatened not to report. In all these cases only one father reported the case to the police and a few ran away. No disciplinary action was taken against any of them.

*A family friend who used to visit my mother and assists me with my homework made me pregnant. One day, he asked me to pay him a visit. I went to his house and he forcefully had sex with me. When my mother threatened to report him to the police, he runway.* (16 years).

*One evening after supper, the woman I was working for informed me that one of the fishermen we work with was looking for me. I protested that it was late and would rather meet him the following morning. But she insisted that I should meet him and that he only wanted to talk to me. So, I went and the man forcefully had sex with me. I came back and informed madam while I was bleeding. She said because it was my first time and told me to keep quiet.* (16 years).

*My uncle asked me to fetch him a bucket of water. I went to his house with my cousin but when I returned, she wasn’t there. Suddenly, he held me from behind, covered my mouth with cloth and had sex with me. That was my first time. This continued. Later, I told my grandmother. Anytime I report she shuts me down and warned me never to mention it again. It was getting too much so I told my friends and cousins. When they told her she beat me mercilessly. My father was angry when he got to know that I’m pregnant and my uncle was responsible. (12 years).*

### Pregnant adolescents’ experiences

Pregnant adolescents’ experiences range from psycho-social, financial, health and educational challenges.

#### Psychosocial challenges

The psychosocial experiences were: denial, sadness, shame with some contemplating abortion and or suicide. They denied initially but accepted the reality later either because of ignorance about the signs of pregnancy, or fear of maltreatment. Their narratives illustrate what happened*:*

*I realised I was pregnant when I missed my period but when my mum asked me, I denied for some time. I was afraid my mother will scold me but I later confessed*. (15 years)

*Immediately I was told the pregnancy was 6 months, I shouted ‘it’s a lie’ because my stomach was its normal size. After that my sister and I bought test kits and did three more tests which were all positive, but I was still in a state of denial for a long time until one day my sister showed me a dark line on my stomach to prove that I was pregnant. That was when I accepted the pregnancy.* (17 years)

Participants whose parents scold and or beat them recounted their experiences as follows:

*When we got home from the clinic after confirming my pregnancy, I received the beatings of my life from my mother. This made me cry for days.* (14 years).

*My mother got angry about the pregnancy and said I am not her daughter. She yells at me and says I’ve disappointed her by getting pregnant. (18 years)*

Participants described how they saw their future shattered with some contemplating abortion and or suicide.

*Hmm! I was very sad when I was told I was pregnant. I cried the whole day. My dreams came crushing down right in front of me. I wanted to go to school and become somebody in future. I told myself if I knew any medicine, I would have terminated the pregnancy. I also contemplated committing suicide by hanging myself with a rope.* (17 years)

*I thought of having an abortion. It was also suggested by a friend who told me she can help me. But I couldn’t do it because we were told in class that when you try to abort a baby, you can die from the procedure. I wasn’t ready to die. (13 years).*

Regarding their partners, only two adolescents accepted responsibility while the rest denied or run away. They described their partners’ behaviour as follows:

*The boy denied the pregnancy and rejected me. He does not provide any support* [crying]*. This makes me cry most of the times I feel like dying. (14 years).*

*My partner denied responsibility but later said we should have abortion when my mother confronted him and his parents. My mother refused aborting the pregnancy, so he no longer talks to me. His parents said they don’t want to be involved and that it’s my fault to have allowed myself to get pregnant (14 years).*

Some participants suffered rejection and were scorned by their peers and neighbours. Their accounts are:

*Some of my friends and children in my area do not want to talk to me. They laugh at me and tell me I’m no longer part of them because I am pregnant. It makes me sad.* (13 years)

*Some women in the area laugh and gossip about me; saying why a little girl like me should get pregnant. They say small girl like me, instead of going to school I’m following boys and ‘penis’ and call me “funorvi”* [pregnant girl]. *They tell my friends to stay away from me because I will spoil* [influence] *them and they will become pregnant.* (14 years).

#### Financial challenges

Almost all the pregnant adolescents studied had financial challenges. Parents stopped providing financial support at the initial stages of the pregnancy. Though parents and two partners finally provide financial support, the girls indicated it was inadequate. The following narratives show the financial challenges pregnant adolescents face:

*The boy who impregnated me rejected me. He does not support me. His mother also doesn’t support me. It is difficult for me. This makes me cry most of the time and I think of dying. (14 years).*

*My mother scolds me and says I have become a burden on her. She tells me she can only give me what I need to take care of the pregnancy. (16 years.)*

#### Health challenges

Almost all participants reported feeling unwell, headache and anaemia. They were told at the ANC that their haemoglobin level was low. They reported their health conditions as follows:

*I easily feel tired and so I am unable to work as I used to. I was told by the nurses that my blood* [haemoglobin] level *was low. The nurses told me to eat well* (13 years).

*I often experience headache. After the laboratory test, the nurses told me I’m anaemic. She said I don’t eat well. She advised me to eat well and take the medicines prescribed for me. (R8, 16 years)*

#### Educational challenges

All the in-school participants stopped going to school when the pregnancy was confirmed. They said:

*I’m a very good student. I cannot register for WASSCE. My dad stopped me from going to school. I didn’t like the idea but I don’t have a choice than to stay home.* (17 years)

*I loved going to school till I got pregnant. I was in the cultural troupe and the first in class. My mother asked me to stop going to school* (15 years).

### Coping with pregnancy

The pregnancies were unwanted, so participants experience psychosocial challenges and developed strategies to cope. In addition to support from parents and other family members, adolescents use avoidance of people, depending on God and prayer to cope with their pregnancy.

#### Support during pregnancy

Generally, parents, other family members and neighbours’ blamed adolescents for getting pregnant and were reluctant to support them initially. However, many of them later provided financial and emotional support, counselled them on how to take care of themselves. Partners who accepted responsibility for the pregnancy were generally supportive*.* These narratives show the support participants received was insufficient:

*My family finally accepted the pregnancy and assisted me. They do not hesitate when I ask for help and advise me on how to eat and take care of myself. They sometimes help me with my chores and accompany me to the ANC. Their support gives me hope and reduce the worry. (15 years).*

*The man responsible for my pregnancy supports me financially and encourages me. He gives me everything I need. He makes me forget my worries. He promised opening a shop for me when the child grows.* (15 years).

*Some elderly women in the neighborhood educate me on how to take care of myself.*

*I have enough support to keep me going (R5, 16 years).*

#### Avoidance of people

Avoidance as a coping strategy is characterised by not going to public places and staying away from people. Pregnant adolescents stopped going to school. While some stopped going to church, others reduced the frequency of attendance, go late and or leave early. Their narratives are:

*I don’t go out; I don’t go to school nor church. In my church when you get pregnant and you are not married, they will call you to the front and tell you to sit at the back. I don’t want to receive that kind of treatment so I stopped attending church. (14 years).*

*Since I got pregnant, I stopped going out because I don’t want people to laugh at me. I stopped going to school. I sometimes go to church but late. I sit at the back and leave early so that many people will not see me. (13 years).*

#### Dependence on God and being prayerful

Dependence on God and prayer helped many adolescents to cope with pregnancy. They explained what they did:

*I’m sorry about the pregnancy but I take solace in God’s words. He said we should call on him when we are in distress and in need and he will be there for us. (19 years).*

*I trust in God. I’m always praying to God to carry me through this pregnancy and help me deliver the baby safely. (14 years).*

## Discussion

This study explored how adolescents get pregnant, their lived experiences, and coping strategies.

### Social factors influencing adolescent pregnancy

Our results echo the evidence that sociodemographic factors such as poverty and low education are strongly associated with adolescent pregnancy [4, 16, 17, 19, 21, 25]. Studies in Accra and Bolgatanga in Ghana found that financial deprivation was a push factor for adolescent pregnancy [16, 21]. Most of the participants in all these studies indicated that they went into sexual relationships to get financial assistance and got pregnant as a result.

Regarding education, fewer adolescents in SHS become pregnant. This supports previous findings that teenage pregnancy is strongly associated with education below secondary level [10, 26]. The GDHSs show that more girls with no formal or primary education got pregnant compared to those who had secondary education [26]. Additionally, majority of pregnant adolescents’ parents had no formal education. Also, mothers and most fathers work in the informal sector. These results suggest that low parental education and informal sector employment, resulting in low paid jobs could influence adolescent pregnancy. These results support the evidence that poverty is a risk factor for adolescent pregnancy.

Finally, none of the pregnant adolescents were married. This contributes to evidence in the GDHS’s report of a declining trend in child marriage in Ghana. The proportion of women married by age 15 declined from 11% in the 45–49 age group to 2% in the 15–19 age group between 1998 and 2014; indicating a declining age at first marriage. However, there was no distinct decreasing trend of teenage pregnancy over the same period [10].

### Sexual exploitation and teen pregnancy

Risky social environment exposed adolescents to sexual abuse especially those aged 12–15 years. Many of them were pressured by family members and acquaintances into having sex while others were lured and defiled or raped by family members, neighbours and acquaintances but none of the perpetuators were punished.

This corroborates previous findings that young adolescents are more vulnerable to sexual abuse [16, 21, 27] as their first sexual experience [28, 29]. Significantly, some adolescents below 16 years who engaged in consensual sex were actually sexually abused as it is not possible for them to give consent. Furthermore, although they agreed, they are not yet at a legal age where they can be in sexual relationships therefore, it is against the laws of Ghana.

It is therefore, worrying that though the 1996 African Charter on the Rights of the Child [29, 30] state that a child below 16 years cannot give consent for sex, only one perpetrator has so far been arrested and the case is still in court after several months. Some adolescents were prevented from talking about and reporting their plight, lest they face the wrath of family members, others could not report because of shame and stigma. These increase girls’ vulnerability, denies them justice and does not deter boys and men from abusing them. Certainly, societal norms have played a substantial role in this problem, where society defers matters of this nature entirely to parents and families of the adolescent girls, No one interferes with the decision of a family not to take up action ostensibly to protect the abused girl from stigma or potentially not getting a husband in the future. It is also considered private to protect the family’s name especially if the perpetuator is a member. The apparent resolve by parents, families and society in general to protect abused girls and perpetuators rather perpetuates of the problem.

### Challenges of pregnant adolescents

#### Health challenges

Headache and anaemia, the main health challenges mentioned by almost all the pregnant adolescents, could be linked to the physiology of pregnancy and financial challenges. As noted by Atuyambe and colleagues in their study, almost half of adolescents experience malaria and anaemia during pregnancy [31]. Lotse also explains that having financial challenges means that the adolescent mother will lack the necessary resources for balance diet and other food supplements [18]. Also, when girls become pregnant, they have limited employment opportunities and are likely to land in poverty and unable to have the needed balanced diet and required nutrition. These often results in adverse pregnancy and birth outcomes such as stillbirth, preterm birth, neonatal death, congenital anomaly, and low birth weight [32].

#### Girls’ education and adolescent pregnancy

All the in-school pregnant adolescents stopped going to school. This result agrees with previous findings that pregnancy truncates girls’ education [16, 27]. However, the result contradicts the finding that school drop-out leads to early sexual activity resulting in adolescent pregnancy [33]. The possible reason could be that Ghana’s efforts in keeping girls in school resulted in many of them progressing beyond primary school. However, as they enter adolescence, pregnancy remains a threat to their retention. Furthermore, when girls become pregnant and drop out of school, they have limited employment opportunities and often land in poverty, which has ripple effects on themselves, family, and the country at large. Therefore, there is the need, not only for parents to monitor and have open sexual conversation with their children, but for school authorities to intensify adolescent school health education programmes that would keep adolescents focused on their academic work and also protect themselves against unwanted pregnancies [21].

Our finding also show that all in-school adolescents stopped going to school immediately their pregnancy became obvious. This contrasts the situation in the United States of America where pregnant African American adolescents are supported and motivated to continue schooling [13].

### Psychosocial challenges of pregnant adolescents

The negative reactions show that pregnancy among adolescents is pathologised. As reported by previous findings, pregnant adolescents experience rejection, stigma and other negative reactions from family members, partners, peers and neighbours because of culture and religion [18, 20, 34, 35]. These result in emotional and mental distress and shame with some having suicidal ideation [27, 36–38]. In this regard, it is important to eschew religious and cultural norms and beliefs that are inimical to the psychosocial development of pregnant adolescents. This problem could be addressed if significant people and institutions in the community accept that adolescents made a mistake by getting pregnant and support them to give birth and return to school.

Furthermore, negative reactions towards pregnant adolescents reveal how gender influence people’s response to teen pregnancy. Instead of pregnancy being a shared obligation, girls are blamed for getting pregnant. Hence, men’s apparent absence in the teen pregnancy discourse illustrates a stigmatising social environment that leads to pregnant adolescents experiencing shame and stigma and more likely to report embarrassment than boys [12, 34, 39].

### Coping strategies of pregnant adolescents

Notwithstanding society frowning upon teen pregnancy and adolescents’ grief, they were averse to abortion. Rather, their social network provided support while adolescents devised strategies to cope. This endorses previous findings that support from social network is critical to adolescents’ ability to cope with pregnancy [28, 38, 40]. Also, our results corroborate previous findings that pregnant adolescents withdraw from unfavorable environment as a buffer to scorning and rebuke—all of them stopped attending school. Some stopped going to church, others go late, sit at the back and leave early while they pray and believe that God will keep them safe [28, 39–42]. These results suggest that churches which are expected to support pregnant adolescents as a vulnerable group are not able to do so quite well.

These coping strategies could help adolescents in several ways. An open parental communication on sexual behaviour issues at home, comprehensive sex education in school and positive attitude, self-efficacy, risk perception towards contraception, alongside with goal-setting, could be protective factors in adolescent girls’ pregnancy prevention efforts [25, 41]. Furthermore, it is recommended that adolescent mothers who return to school adopted conscious avoidance of incisive remarks, vicarious experience as well as self-determination as coping strategies [43].

### Limitations of the study

﻿This study was conducted in only one district and information gathered could not be verified from partners and parents. However, since participants were selected from communities across the district, diverse groups of pregnant adolescents could be represented. Therefore, the findings should be applied with circumspection.

### Implications for policy and practice

The Ghana Education Service (GES), the Domestic Violence and Victims Support Unit (DOVVSU) of the Ghana Police Service, Judicial Service and Social Welfare Department should collaborate with schools, communities and religious institutions to seek justice for abused pregnant adolescents, provide shelters for those whose families might want to pervert justice, help them continue schooling after delivery and parents should provide girls’ basic needs to prevent their dependence on boys and men. Although the use of contraceptives is common among Ghanaian adolescents, this has been declining from 22.1% in 2003 to 20.4% in 2014 [44]. This may be due partly to societal norms that do not encourage contraceptive use among unmarried adolescents who are expected to abstain from sex [45]. Therefore, it is important for parents, families and the society generally to encourage the use of contraceptives among adolescents. Also, the decline in contraceptive use among adolescents may be due to lack of access or inability to obtain them because of the stigma associated with going for it. It is therefore imperative for parents, families and the society to start having conversations that would lead to encouraging and motiving adolescent girls to use contraceptives. It is known that as adolescents in basic schools are becoming sexually active, there is a need for formalised contraceptive education in basic schools for correct information and education [46] in addition to abstinence which has been traditionally promoted but can no more be guaranteed as girls’ risk of engaging in unprotected sex increases.

## Conclusions

The results of this study show that adolescent pregnancy occurred through consensual sex, transactional sex and sexual abuse. Despite parents’ harsh treatment and adolescents’ anger, both were averse to induced abortion. While parents provide support, pregnant adolescents self-isolate, depend on God and pray to cope with pregnancy.

Considering the implications of pregnancy for girls’ well-being, the GES, the DOVVSU and Judicial Service should collaborate with schools, communities and religious institutions to identify situations that expose girls to transactional sex and sexual abuse and provide early intervention, detect sexual abuse cases and seek justice for victims to deter perpetrators. Also, Social Welfare Department should be resourced to provide shelters for pregnant adolescents whose families might want to pervert justice and help them continue schooling after delivery. Finally, we suggest a larger study that will engage parents, partners and community leaders.

## Data Availability

The data are not publicly available at the moment due to confidentiality issues but can be obtained from the corresponding author on reasonable request.
